# A camphene-camphor-polymer composite material for the production of superhydrophobic absorbent microporous foams

**DOI:** 10.1038/s41598-021-04240-5

**Published:** 2022-01-07

**Authors:** Richard J. G. Löffler, Martin M. Hanczyc, Jerzy Gorecki

**Affiliations:** 1 Institute of Physical Chemistry, Polish Academy of Sciences, Kasprzaka 44/52, 01-224 Warsaw, Mazowieckie Poland; 2 Laboratory for Artificial Biology, Department of Cellular, Computational and Integrative Biology (CIBIO), University of Trento, Polo Scientifico e Tecnologico Fabio Ferrari, Polo B, Via Sommarive 9, Povo, 38123 Trentino Alto-Adige, Italy; 3 Farris Engineering Center, Chemical and Biological Engineering, University of New Mexico, Albuquerque, NM 87106 USA

**Keywords:** Chemical physics, Surfaces, interfaces and thin films, Chemical physics, Surfaces, interfaces and thin films

## Abstract

In a recently published paper (doi.org/10.3390/molecules26113116) on self-propelled motion of objects on the water surface, we described a novel surface-active plastic material obtained by dissolution of camphor and polypropylene in camphene at 250 $^\circ$C. The material has wax-like mechanical properties, can be easily formed to any moldable shape, and allows for longer and more stable self-propelled motion if compared with pure camphor or pure camphene or of a camphene-camphor wax. Here we use scanning electron microscopy to visualize and characterize the microporous structure of the solid polypropylene foam formed in the plastic for different polypropylene contents. The topology of foams remaining in the material after camphor and camphene molecules have been removed through evaporation or dissolution is similar to polypropylene foams obtained using thermally-induced phase separation. We show that the foams have a superhydrophobic surface but strongly absorb non-polar liquids, and suggest an array of potential scientific and industrial applications.

## Introduction

Polymer solid foams are important materials widely used in modern technology. Their applications range from cushioning^1^ and insulation^2^ to sophisticated microporous environments with controllable mechanical behavior and structures^3–6^. Such structured environments are especially useful in catalysis^7–11^, filtering (as for example membranes)^12–14^ and as highly efficient absorbents^12^, such as aerogels made of various functionalized and composite materials^15–19^.

A solid foam can be defined as a uniform distribution of air and solid phase in a certain volume. Polymer foams are typically produced by combining a polymerization or curing reaction with a reaction that produces gas (oftentimes one and the same reaction). Other methods include the use of porogens, which are substances evenly distributed in a polymer before leaching to leave behind a porous polymer lattice. Such porogens can be supercritical CO_2_^20,21^ or substances that are co-soluble in the polymer and can be removed by dissolution or thermally-induced phase separation^22,23^. One group of substances that has been recently applied for such purposes are terpenes which contain chemicals such as camphor, camphene or eukalyptol. Terpenes offer a range of physical (volatility), chemical (co-solubility, plasticizing) and mechanical (crystalline, waxy or liquid) properties that are important for applied porogens. There are several papers reporting on the use of either camphor or camphene as porogens especially in thermally-induced phase separation (TIPS) for the production of microporous membranes^13,22–25^. In the majority of reported cases the terpene content was low compared to the polymer content leading to dense porous materials. Combinations of camphor and camphene have only been used as porogens for polymer-ceramic composite materials^26,27^.

Our interest in polypropylene foams was initiated through research on self-propelled materials that could be easily formed into arbitrary shapes. Such materials are important for experiments illustrating and elucidating relationships between the geometrical shape of a solid, self-propelled object and the character of its motion on the water surface^28–33^. While developing a wax-like self-propelled material we discovered the miscibility of camphor and camphene with thermopolymers such as polypropylene or polyethylene^34^. The presence of polypropylene improved the usefulness of material in experiments if compared to a camphor-camphene wax, described in^35^. We observed that polypropylene stabilized the speed of self-propelled objects in time. Moreover, unlike for camphor-camphene waxes, the motion of objects made of camphene-camphor-polypropylene plastics hardly depends on the weight ratio between camphor and camphene. When the motion of an object terminates and the surface active components (camphor and camphene) fully dissipate, the plastic transforms into a soft and lightweight microporous foam. We speculated^34^ that the increase in stability results from the presence of polypropylene scaffold that forms in the molten plastic and filled up the object. The presence of such a scaffold moderates the release of surface active molecules.

In this publication we report the qualitative characterization of the microporous structures in polypropylene foams that are formed in camphene-camphor-polypropylene plastics. We demonstrate how the volatile components can be removed and describe some potentially useful characteristics of the polypropylene foams. Furthermore, we discuss directions for future studies on the foams and point out possible applications. To our knowledge, a bulk foam with the properties presented here has not previously been produced using an optimized hybrid camphene-camphor porogen in combination with low concentrations of polymer.

## Experimental section

The production protocol of camphene-camphor-polypropylene plastic was described in our recent paper focused on self-propelled motion^34^. The plastic was made of a mixture of components: camphor (98% purity, CAS: 464-49-3, Sigma-Aldrich), camphene (95% purity, CAS: 79-92-5, Sigma Aldrich) and polypropylene in the form of pellets (CAS:9003-07-0, Sigma Aldrich, product number 427861, number average molecular weight $M_n \sim 97,000$ and weight average molecular weight $M_w \sim 340,000$). The components were added to a beaker along with a magnetic stirrer and placed on a hot plate set to 250$^\circ$C. We dissolved 5 or 10% (higher concentrations are possible) of polypropylene by weight in a liquid camphene-camphor mixture at temperatures below 250 °C. Therefore, in our experiments the polymer is dissolved in the terpene mixture instead of the other way around as is described in other publications^22^.

For the production of foam samples used in this publication, liquid camphene-camphor-polypropylene plastic was poured into a receptacle, lined with a nitrile or latex rubber sheet. Once the plastic cooled to room temperature, one could cut it with a scalpel into samples or shape it by hand into the desired form. Rod-shaped foams can be obtained by extruding the camphene-camphor-polypropylene plastic (or other precursor material) through a heated syringe or using high pressure extrusion. Furthermore, this method can be applied using other thermoplastic polyolefins such as polyethylene or polyisobutylene.

To obtain a foam, the porogens (camphene and camphor) can be removed passively through evaporation, which is a slow process and can take several days depending on the size of the sample. To accelerate it one can use a solvent such as ethanol or acetone. This was done by immersing a sample of the camphor-camphene-polypropylene plastic in the solvent of choice for a few minutes before removing it from the solvent and letting any remaining solvent evaporate from the sample. The evaporation can be accelerated using a blow dryer or a similar tool. This removal step was repeated with fresh solvents three to four times until all of the camphor and camphene had been removed. Preferably the sample is then left under ventilation overnight to make sure that all solvent and remaining camphor and camphene have evaporated from the foam. This procedure yields a microporous, hydrophobic, lipophilic polypropylene foam. Rod-shaped foams can be obtained by extruding the camphene-camphor-polypropylene plastic (or other precursor material) through a heated syringe or using high pressure extrusion. Furthermore, this method can be applied using other thermoplastic polyolefins such as polyethylene or polyisobutylene.

For the characterization of the porous structure, the polypropylene foam samples were investigated under a high magnification using a Zeiss FESEM-Supra 40 field emission scanning electron microscope. The foams of two different compositions were cut into small cubes, mounted to the specimen mount using conductive carbon adhesive tape, placed in the plasma sputter coater and coated with a thin layer of platinum. The prepared samples were then imaged at different moderate magnifications (up to 3kX).

The mechanical stability of the foams was tested on cubes of two different foams (5% polypropylene and 10% polypropylene) and exposing them to increasing weights of up to 1200g. The deformation was documented by taking pictures of the samples before and after application of the weights.

For the initial contact angle measurements, a rectangular foam sample was prepared as a $\sim$1 × 1 cm square with the thickness of $\sim$2mm. Droplets of pure water with different volumes (source: Millipore ELIX5 system) were placed onto samples. Contact angle measurements as well as contact angle hysteresis were performed using a Biolin Scientific Theta Life pendant droplet tensiometer with gauge 30 capillary. For the contact angle hysteresis measurements, the capillary was inserted to the droplet and the contact angle was measure while increasing and decreasing the volume of the droplet. Droplet impact was recorded using the same instrument at 220 frames per second.

The absorbent properties of the material were demonstrated by adding a 200 μl droplet of paraffin oil (puriss.,Viscous Liquid, EC Number: 232-384-2, Sigma-Aldrich) stained with 0.5 mg/mL Oil red O (BioReagent, Sigma Aldrich) to a pure water surface in a 9 cm Petri dish. A foam sample made from 45% camphor, 45% camphene and 10% polypropylene precursor mixture with the dimensions 0.5 × 1 × 1.5 cm was then placed adjacent to the droplet and the absorption was filmed from above using a mounted digital camera (NEX VG20EH, SONY) while being illuminated from below for better contrast (except the last few frames to demonstrate the color change of the foam due to the absorbtion of the dyed oil).

The absorption efficiency of foams made with 5 and 10% polypropylene was measured by producing a series of cubes from these materials and immersing them in paraffin oil for over two minutes in order to ensure full absorption. The cubes were weighed before ($m_0$) and after (*m*) immersion and the dimensionless absorption efficiency (*Q*) was calculated using the simple formula:

1 $$\begin{aligned} Q=\frac{(m_0-m)}{m_0} \end{aligned}$$

## Results and discussion

Foams of diverse shapes can be easily made with the malleable precursor plastic material. We found that the malleability of the precursor material previous to removal of the porogen mixture can be adjusted by changing the weight ratio between camphor and camphene. Higher camphene contents improved the malleability of the plastic, but in turn increased the precursor plastic material stickiness. Malleable precursor plastic material enabled the production of foam samples with complex shapes.

**Figure 1:**
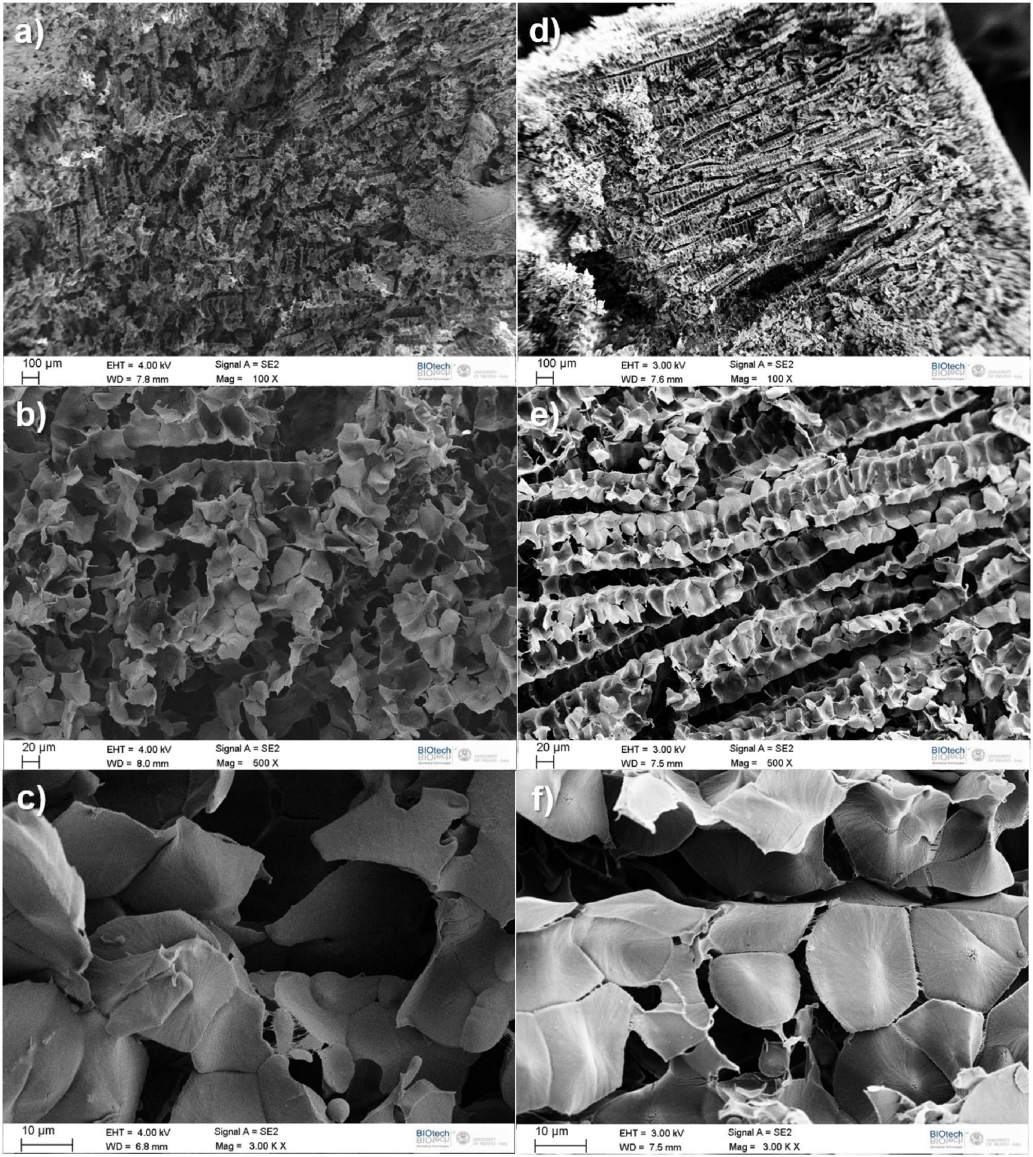
Scanning electron microscopy (SEM) images of the polypropylene foam structures obtained from the plastic produced with $47.5 \%$ weight ratio of camphene and camphor and $5 \%$ weight ratio of polypropylene (**a**, **b**, **c**) with the foam made using the plastic containing $45 \%$ weight ratio of camphene and camphor and $10 \%$ weight ratio of polypropylene (**d**, **e**, **f**). The magnifications were 100X (**a**, **d**), 500X (**b**, **e**) and 3000X (**c**, **f**).

**Figure 2:**
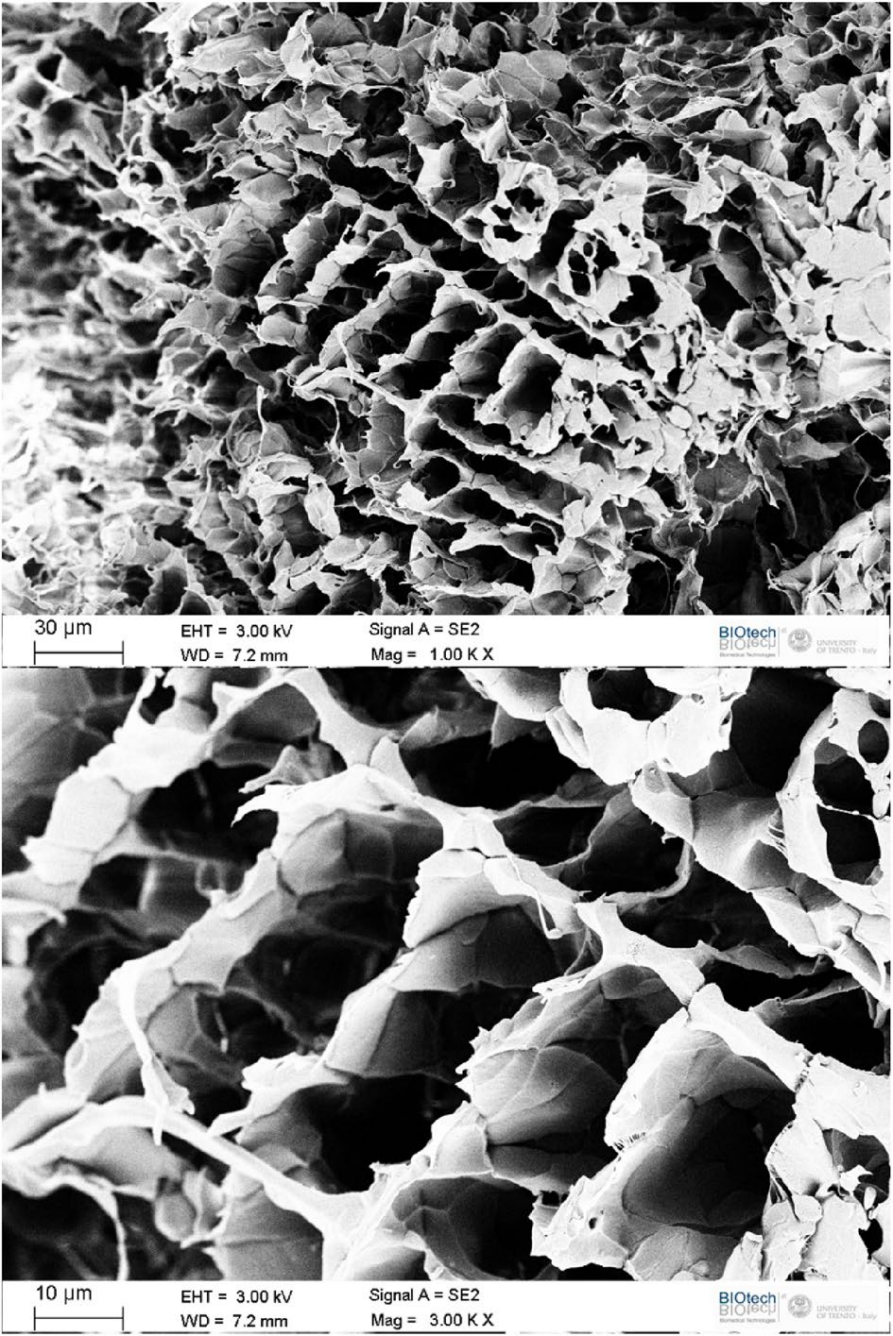
High magnification SEM micrograph of a sample made with 10% polypropylene showing a close-up of the ordered structure made from flakes and spine-like columns.

In Fig. 1 we compare scanning electron microscopy (SEM) images of the polypropylene foams obtained from the plastic $47.5 \%$ weight ratio of camphene and camphor and $5 \%$ weight ratio of polypropylene (Fig. 1a–c) with the foam made using the plastic containing $45 \%$ weight ratio of camphene and camphor and $10 \%$ weight ratio of polypropylene (Fig. 1d–f). For both component ratios we present images at three different magnifications (100X, 500X and 3000X). It can be seen that foams made as described in the "Experimental section" retain organized microscopic porous structures. We observe columns of polypropylene made of pores separated by regularly distributed walls. The pore size decreases with the increasing polypropylene concentration in the mixture as the foam becomes slightly denser (cf. Fig. 1b,e). For both 5% and 10% the pores are less than 30 μm long in the column direction and less than 50 μm wide in direction perpendicular to the column axis. High magnification SEM images of columns and pores obtained from a sample with 10% polypropylene are illustrated in Fig. 2. Similar pore structures were observed for much higher polypropylene content (24 %) in membranes produced by thermally induced phase separation (cf. Fig. 8 in^22^).

The comparison between Figs. 1a and 1d indicate that, more ordered structure of columns appear when the polypropylene concentration was increased from 5 to 10%. For 5% of polypropylene columns are shorter and aligned at short distances only. On the other hand, if the plastic contained initially 10% of polypropylene the columns are longer and the correlation length between their directions is much longer as well. The observed effect seems similar to the density driven transition between weakly ordered liquid crystal and the nematic phase.

The pore walls are not uniform, but they are formed of polypropylene flakes. At yet higher magnifications some of the structures appear to be in the nanoscopic regime with the flakes having a thickness significantly smaller than their diameter (ca 5–20 μm in diameter and about 0.5–2 μm in thickness, See Fig. 1c, f. For 5% of polypropylene the arrangement of individual segments is disordered with large and thin flakes (Fig. 1c). For 10% polypropylene, the sizes of the individual flakes are slightly smaller but, in general, thicker with a higher order organized structure (See Figs. 1f and Fig. 2).

**Figure 3:**
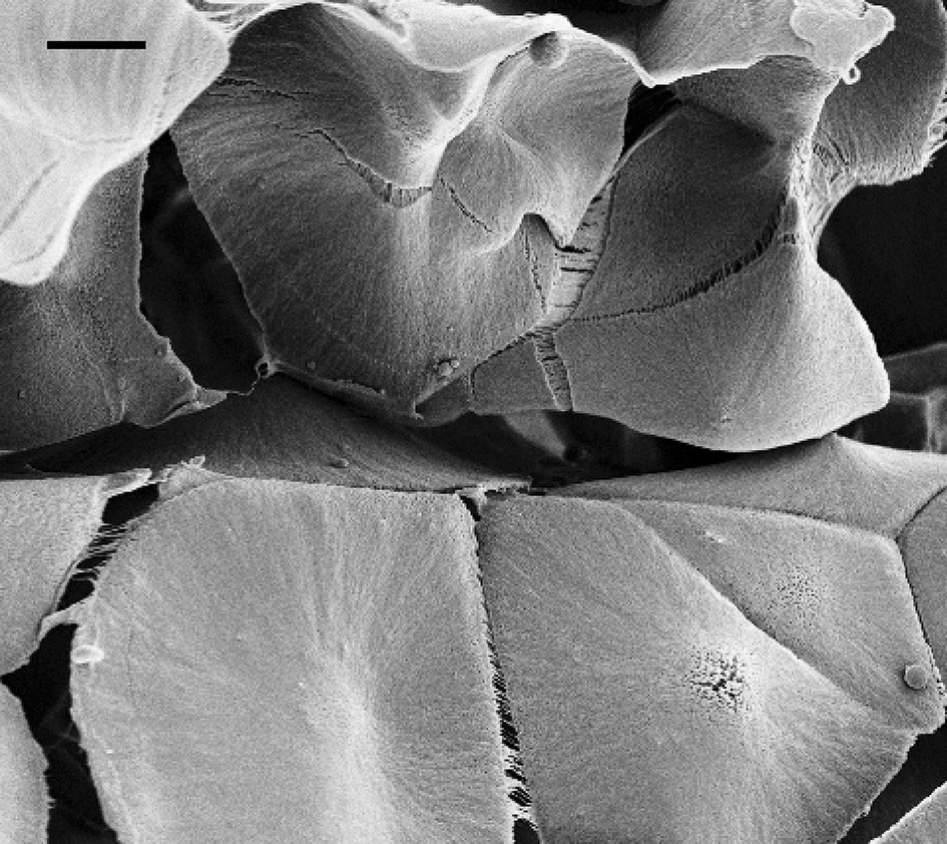
High magnification SEM micrograph of a sample made with 10% Polypropylene showing polymer flakes attached to each other by very thin fibres, presumably in the nanometer scale. Scale bar = 5 μm.

The ordered structures of polypropylene foams resemble insect hives with evenly distributed pores and a scaffold (cf. Fig. 2). At very high resolutions one can see that flakes are interconnected and stabilized by thin fibrils connecting them (cf. Fig. 3), which appear to be only tens of nanometers thick. The ordered structure may result from spinodal decomposition with the ordering taking place, most likely during the solidification of the precursor polymer-wax^36^. Alternatively, the spinodal channels could be formed as evaporation/dissolution pathways during the removal of the camphene-camphor porogen mixture.

**Figure 4:**
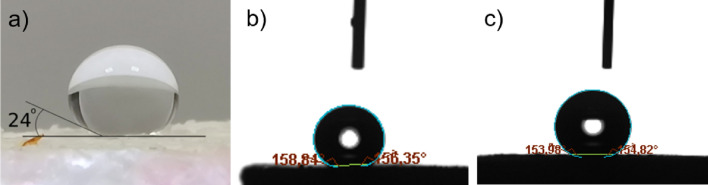
(**a**)100 μl droplet of water was placed on a polypropylene foam sample with the rough porous surface exposed. It can be observed that the contact angle between the droplet and the foam surface is larger than 150$^\circ$. The white cap of the water droplet is merely the reflection of the laboratory ceiling. The instantaneous contact angles measured for droplets with volumes of: 2 μl (**b**) and 5.8 μl using the tensiometer. The needle gauge of the capillary seen above the droplet in (**b**) and (**c**) is 30 (internal diameter of 0.16 mm).

The obtained polypropylene foams are strongly hydrophobic. Figure 4a illustrates a (100 μl) water droplet placed onto the surface of a foam obtained from a plastic containing 10 % of polypropylene. The estimated contact angle ( over 150 degrees ) falls into the superhydrophobic range^37^. Due to the uneven and porous surface along with the innate hydrophobicity of polypropylene the material gains the lotus effect-like water repellency^38^. The estimation of contact angle for a water droplet was confirmed by more precise measurements for small droplets: 2 μl in Fig. 4b and 5.8 μl in Fig. 4c. The average values of contact angles, measured over several seconds, and their dispersions were: $161.9\pm 5.6$ deg and $154.7\pm 1$ deg for 2 μl and 5.8 μl respectively. The differences in contact angles can be attributed to various structures of the surface.

**Figure 5:**
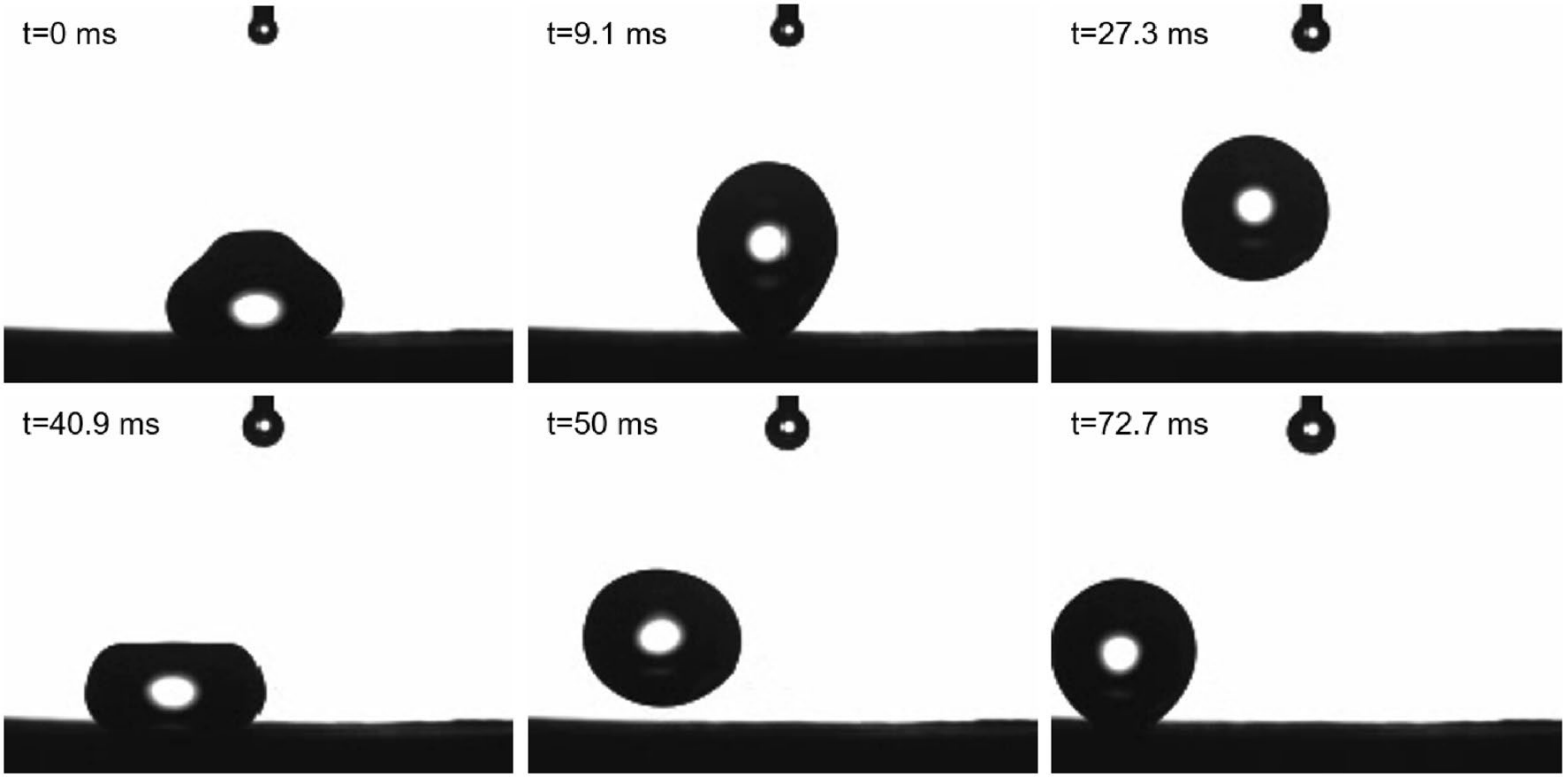
Snapshots form the movie droplet-impact-test.avi enclosed in the supplementary material. The corresponding times are given in the upper left corner of frames.

A high hydrophobicity of the foam can be seen in a 6 μl water droplet impact test^39^. Figure 5 and the movie droplet-impact-test.avi enclosed in the supplementary material show the time evolution of a droplet dropped on the foam surface. One can see that after impact the droplet is reflected without visible decrease in size and with a large fraction of pre-impact energy. The bouncing continues for over a 0.1 s.

**Figure 6:**
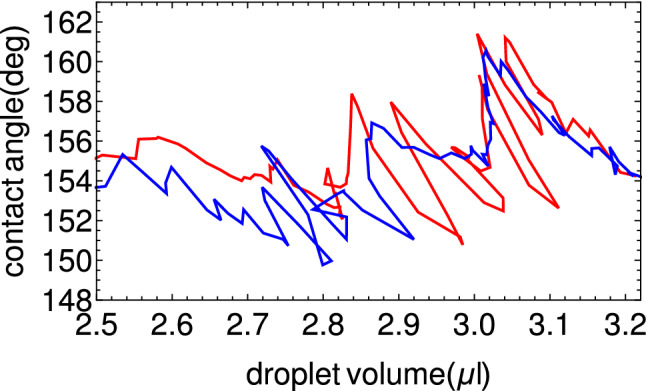
The contact angle measured in an experiment with time-dependent droplet volume; the red and blue curves were obtained for increasing and decreasing volumes at the rate 0.1 μl/s.

We have also measured the contact angle for a water droplet with increasing and decreasing volume (cf. Fig. 6) The red and blue curves correspond to the phases of experiment in which the droplet volume was increasing or decreasing respectively at the constant rate 0.1 μl/s. A little difference between the advancing and receding contact angle can be seen but the hysteresis is small^40^.

**Figure 7:**
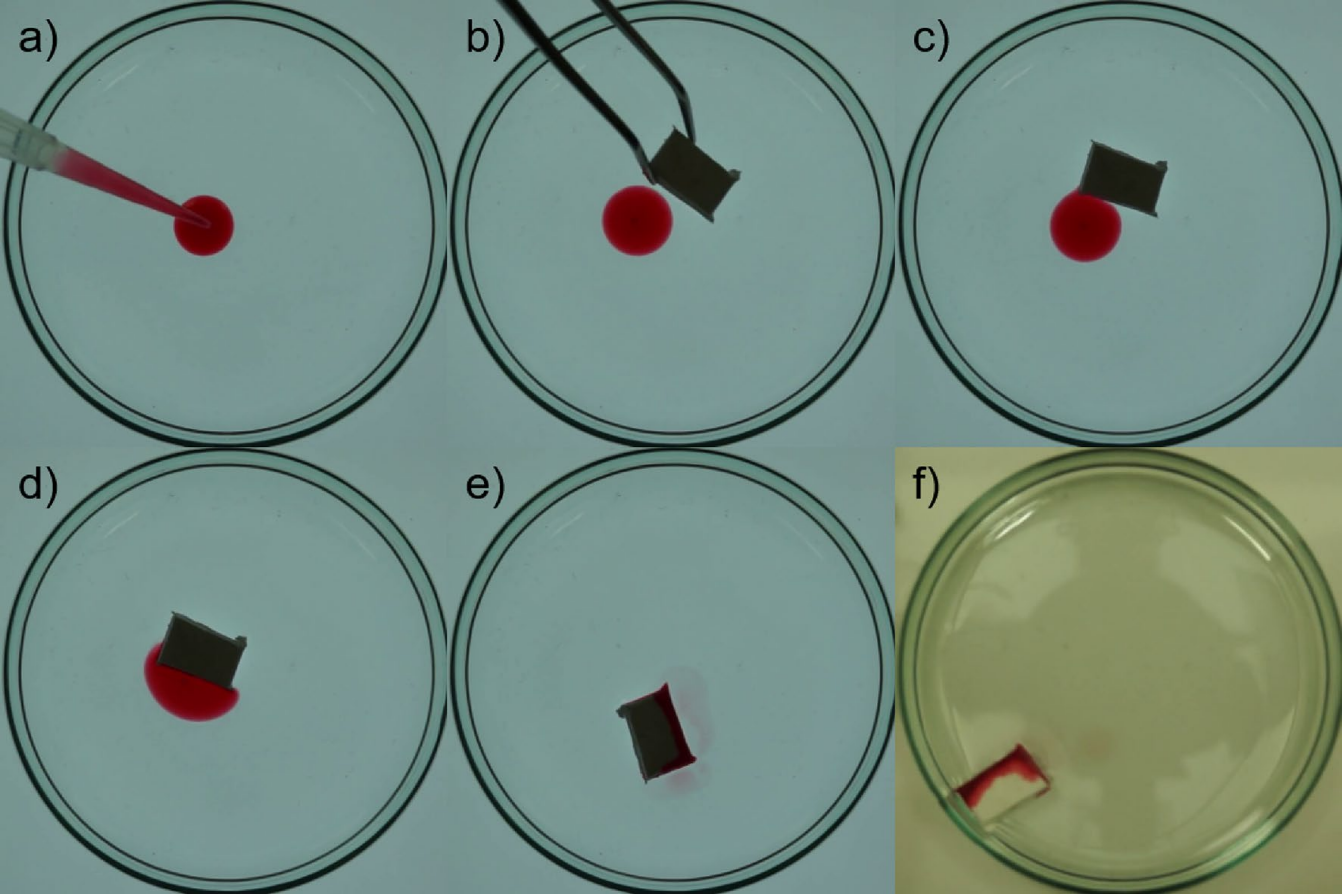
A 200 μl droplet of paraffin oil stained with the dye Oil red O added to a water surface inside a 9 cm Petri dish was absorbed within 4 minutes by a polypropylene foam with the dimensions $0.5 \times 1 \times 1.5$ cm. The foam was made from 45% camphor, 45% camphene and 10% polypropylene mixture and camphor and camphene were removed completely. Frames (**a**–**e**) are back lit for better contrast and show snapshots of “foam-abs.avi” (See SI) at progressing non-even time intervals. Frame (**f**) was taken at the end of the experiment to show that the oil along with the stain has been absorbed by the foam sample.

Since foam samples are highly water repellent, they have a high affinity towards non-polar liquids such as oils or solvents. This property seems especially useful for the removal of oils from water surfaces, as is demonstrated in Fig. 7 and in the movie “foam-abs.avi” (see SI). In this movie a 200 μl droplet of paraffin oil (a mixture of different length alkanes that presents as a slightly viscous liquid with the density $\rho _{para} \sim 0.85$ g/ml) stained with the dye Oil red O was added to a water surface inside a 9 cm Petri dish. A foam sample was made from 45% camphor, 45% camphene and 10% polypropylene mixture after the camphor and camphene were completely removed. The piece of the foam with the dimensions 0.5 × 1 × 1.5 cm was placed on the water surface. Frames a) to e) are back lit for better contrast and show snapshots of “foam-abs.avi” at progressing, non-even time intervals. The frame f) was taken at the end of the experiment to show that the oil along with the stain had been absorbed by the foam sample. Within four minutes the foam sample was able to almost completely absorb all of the oil from the surface and can be removed along with it. Furthermore, it is visible in Fig. 7f that the foam is not fully saturated.

Porosity describes the fraction of void space in the material. It is a parameter that characterizes porous materials like foams^41^. It is defined as:

2 $$\begin{aligned} \phi = \frac{V_{V}}{V_{T}} \end{aligned}$$

where $V_V$ is the volume of void-space available for paraffin and $V_T$ is the total volume of material, including the polypropylene and void components.

For both types of foams discussed here, porosity was obtained by immersing several cube-shaped samples in pure paraffin oil for several minutes and recording the masses before and after immersion. In our experiments we measured the mass of polypropylene foam $m_{polyp}$ after camphor and camphene were removed and the mass of the foam saturated with paraffin oil $m_{para}$. Knowing the specific mass of polypropylene $\rho _{polyp} = 0.9$ g/ml and of paraffin oil $\rho _{para}$ we obtain $V_V = m_{para}/\rho _{para}$ and $V_T = V_V + m_{polyp}/\rho _{polyp}$.

We performed a number of experiments for samples of different volumes and obtained $\phi _5= 0.896 \pm 0.010$ *and* $\phi _{10}= 0.861 \pm 0.016$ as the average porosity for foams obtained using 5% and 10 % of polypropylene, respectively. The absorption efficiency *Q* measured as the ratio between the mass of paraffin and the mass of polypropylene foam was: $Q_5 = 8.2 \pm 0.82$ and $Q_{10} = 5.9 \pm 0.79$ for foams obtained using 5% and 10 % of polypropylene, respectively.

Potentially, one could recover part of the oil by squeezing it out of the foam sample in which case the foam will be elastically deformed (i.e. it will not return to its original shape), crushing the porous internal structure. Another way of recovering the foam as well as the absorbate would be to find an appropriate solvent to extract it. We will explore the re-usability of this material in the future.

**Figure 8:**
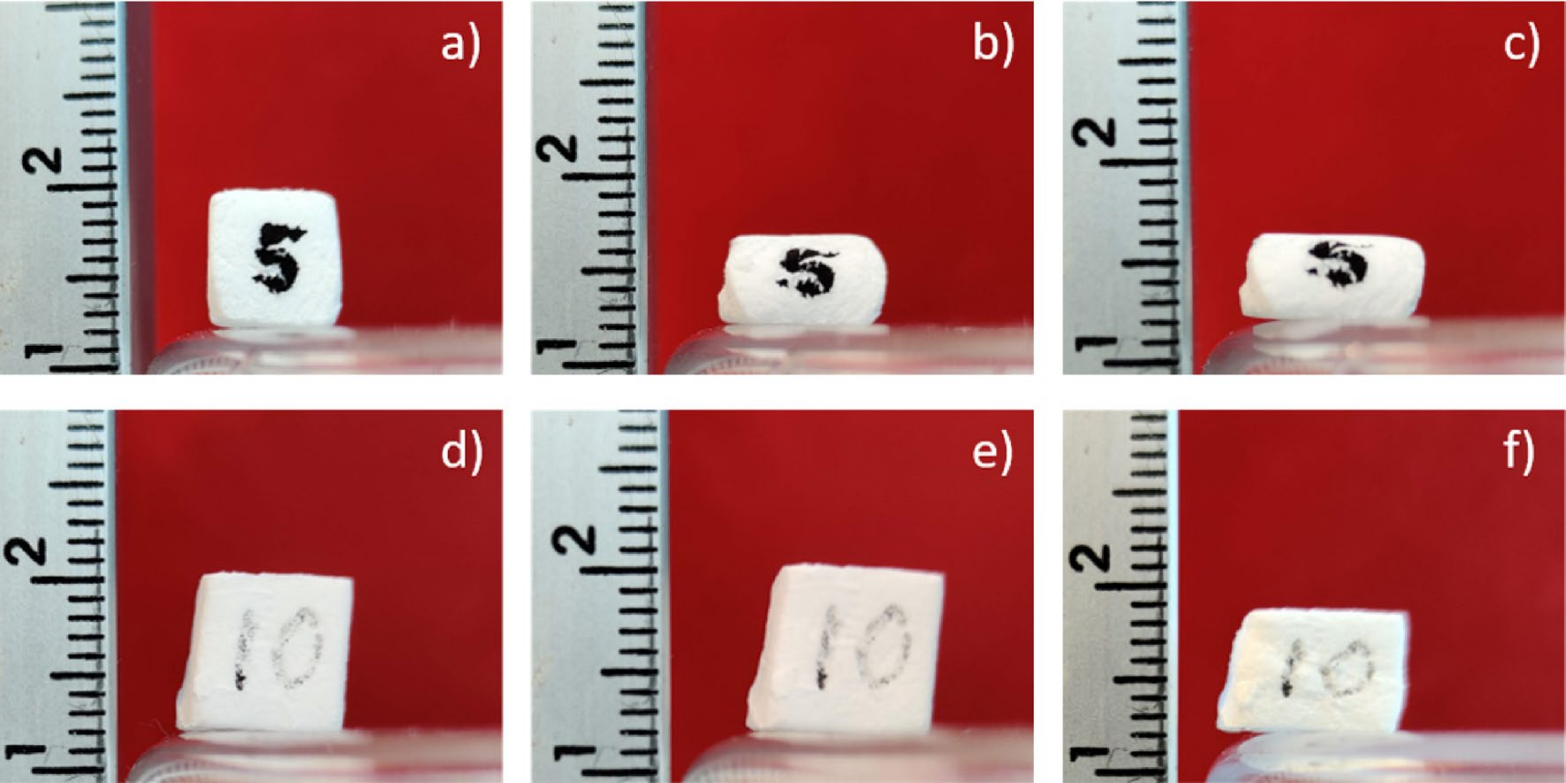
Cube-shaped foam samples made with either 5 or 10% polypropylene were exposed to increasing weights and photographs before and after the application. For the 5% sample the initial sample is depicted in (**a**). In (**b**), the sample was exposed once to a weight of 200g. In (**c**) the sample was exposed to the same 200 g one additional time. It can be seen that the sample strongly deforms after the first application with a small additional deformation after the second application. In the second row of the figure a 10% polypropylene sample (**d**) is depicted after being exposed to 200 g twice in (**e**) and after exposure to 1200 g in (**f**). It can be seen that the increase in polypropylene density results in a significantly increased stability.

The mechanical stability of the foam depends on the amount of polypropylene added to the precursor material. The 5% polypropylene samples were considerably less stable than the 10% polypropylene samples, as can be seen in Fig. 8 where in the top row a cube made with 5% polypropylene (a) was exposed to a weight of 200 g in (b) and once more to the same weight in (c). It can be seen that after the application of a 200 g weight the sample deforms considerably with a further slight deformation after the second application of the weight. In the bottom row of Fig. 8 a sample made with 10% polypropylene (d) did not display a visible deformation after application of 200 g twice (e). A slight deformation of the sample is visible only after application of 1200 g (f). Prior to the application of 1200 g, the sample was also exposed to a weight of 500 g which did not result in any deformation.

## Conclusion

We have investigated the polypropylene structure formed in camphene-camphor-polypropylene plastic we have invented to study self-propelled motion^34^. Through SEM-imaging we found that the polypropylene scaffold formed in objects made of the plastic is indeed microporous and ordered.

We have presented a fast, simple and cheap method to produce a microporous polypropylene foams in bulk with an ordered internal structure. Our study indicates that the structure can be influenced by the component ratio. We have demonstrated that such foams have very useful properties such as superhydrophobicity and the effective absorbance of non-polar liquids (e.g. paraffin oil). Such superhydrophobicity has also been observed with aligned polystyrene nanotube films^42^ and fractal structures^43^. This material can be applicable for the filtering and cleaning of water contaminated with non-polar solvents or similar substances. Other possible applications include thermal insulation materials^2^, catalysts for the chemical industry^11^ and air filtration^14^.

One technological advantage of the material is that its production involves readily available substances, which possibly could be recovered during the production, making it a green process with potentially little to no waste products. The hardness of camphene-camphor-polypropylene plastic strongly depends on temperature, and at temperatures close to the melting point of camphene (ca. 52$^\circ$C) it becomes soft enough to be extruded. The required shapes of plastic, like rods for experiments with self-propelled objects of non-trivial shapes (cf. Figs. 16 and 17 in^34^), can be formed using extrusion. Such thin extruded filaments potentially could be used as a filament in 3D-printers to fabricate pre-determined shapes. The same method can be applied to make polypropylene foams with required shapes.

We plan to continue and extend our study on the polymer foams. Similarly ordered foam structures have been produced with other methods, using higher polymer contents^22,23^. The optical microscopy of the precursor camphor-camphene-polypropylene plastic may reveal whether the porous polymer scaffold is formed during solidification or during the removal of the camphene-camphor porogen. Our recent experiments in which the precursor plastic material was prepared with short chain-length polymers (for example using polyethylene with average M$_w$ $\sim$ 4000 by GPC, average M$_n$ $\sim$ 1700 by GPC (Sigma-Aldrich, CAS: 9002-88-4) produced a powdery substance which crumbles easily after the removal of the camphor-camphene porogen mixture. This observation indicates the importance of using polymers with sufficient chain length.

Besides the obvious technological applications, the described material can be useful for basic research on the relationship between the geometry of confining walls and topology of structures that appear in such confinement. The fact that such correlations do exists have been pointed out in recent papers on structures formed by particles interacting with short-range-attraction-long range-repulsion (SARL) potential^44,45^. The plastics can be easily confined in narrow capillaries with diameters below 100 μm, and we can expect that structures of polypropylene scaffold will depend on the capillary diameter.

Lastly, back our original interest from which the discovery of the presented material came, the foam can be used for very reproducible experiments of self-propelled shapes on water surfaces. As the foam readily absorbs non-polar liquids including surface active substances such as ethanol, decanol or ethyl-salicylate, samples of pre-determined shapes can be “loaded” with the substance of choice and placed onto an aqueous surface. The malleability along with the simple production method enables experiments with multiple equal shapes or mixtures which could act as a macroscopic model system for the study of dissipative self-organization.

## Supplementary Information

Supplementary Information 1.

Supplementary Information 2.
